# Monitoring In Vitro Extracellular Matrix Protein Conformations in the Presence of Biomimetic Bone-Regeneration Scaffolds Using Functionalized Gold-Edge-Coated Triangular Silver Nanoparticles

**DOI:** 10.3390/nano13010057

**Published:** 2022-12-23

**Authors:** Laura G. Rodriguez Barroso, Farah Alwani Azaman, Robert Pogue, Declan Devine, Margaret Brennan Fournet

**Affiliations:** 1 Technological University of the Shannon: Midlands Midwest, Dublin Rd., N37 HD68 Athlone, Co. Westmeath, Ireland; 2 Universidade Católica de Brasília, Campus Asa Norte. SGAN Módulo B 916 Avenida W5—Asa Norte, Brasilia 70790-160-DF, Brazil

**Keywords:** triangular silver nanoparticles, fibronectin, LSPR, extracellular matrix, regeneration scaffold

## Abstract

In the cellular environment, high noise levels, such as fluctuations in biochemical reactions, protein variability, molecular diffusion, cell-to-cell contact, and pH, can both mediate and interfere with cellular functions. In this work, gold edge-coated triangular silver nanoparticles (AuTSNP) were validated as a promising new tool to indicate protein conformational transitions in cultured cells and to monitor essential protein activity in the presence of an optimized bone biomimetic chitosan-based scaffold whose rational design mimics the ECM as a natural scaffold. A chitosan-based scaffold formulation with hydroxyapatite (CS/HAp) was selected due to its promising features for orthopedic applications, including combined high mechanical strength biocompatibility and biodegradability. Functionalized AuTSNP-based tests with the model ECM protein, fibronectin (Fn), illustrate that the protein interactions can be clearly sensed over time through the local surface plasmon resonance (LSPR) technique. This demonstrates that AuTNSP are a powerful tool to detect protein conformational activity in the presence of biomimetic bone tissue regeneration scaffolds within a cellular environment that comprises a diversity of molecular cues.

## 1. Introduction

The crowded extracellular matrix (ECM) controls protein dynamics and trajectories, which underpin important biological processes that can activate response chains for the development of numerous human disorders as well as healing processes. Fluorescence Resonance Energy Transfer (FRET) and Raman Spectroscopy are conventional techniques used to characterize and analyze these protein activities and interactions; however, they are elaborated, and their signals are hindered or obstructed by the high background noise of physiological environments. As an alternative, noble metal nanoparticles are known to have remarkable optical properties and have been intensely researched for the development of highly sensitive nano biosensors to investigate a range of molecules and detect their interactions in the extracellular matrix. Proteins are the most abundant macromolecules in all organisms, with defined amino acid geometries and structures. Three-dimensional protein structures are diverse, ranging from fibrous to globular, and are integral to protein function [1,2]. These structures may possess moving parts where mechanical actions are correlated with biochemical reactions [3]. This correlation between protein conformation and function is being widely analyzed to progress our understanding of biological processes ranging from proteinopathy (protein conformational disorders) development to healing processes [4].

Several techniques exist for the detection of conformational changes and structural variations in proteins, though each has associated limitations or requires compromise for their implementation. These techniques include spectroscopic methods such as Raman spectroscopy, fluorescent methods such as fluorescence resonance energy transfer (FRET), and nuclear magnetic resonance (NMR) [5]. While sensitive when used for protein analysis, they are however remarkably complex, requiring specialized equipment, and their signals are generally greatly hindered by the high background noise of the cellular environment. This complex environment contains water molecules, ions, and metabolites, as well as macromolecules, including proteins, carbohydrates, nucleic acids, and lipids [6]. This represents a challenge when measurements are performed under cellular conditions and required for advanced real-time detection in applications such as personalized medicine [7]. In this context, noble metal nanoparticles present exceptional optical properties which are highly localized at the nanoscale and exhibit important features for the development of highly sensitive nano biosensors to study a variety of molecules and their interactions [8]. Several nanostructures with high refractive index sensitivities have been reported, such as triangular silver nanoparticles (TSNP), silver nanoprisms, nanorice, among others [9]. The strong localization of the detection exhibited by high refractive index sensitive nanostructure provides a critical advantage when operating within high noise environments. For biosensing applications, the local surface plasmon resonance (LSPR) of the nanostructures governs their optical spectrum, which can respond to interactions at the nanostructures’ surface. In this optical phenomenon, light wavelengths which are resonant with the surface electrons result in an extinction spectrum with distinctive LSPR bands [10]. The LSPR position depends on the nanostructures’ shape, size, uniformity, and composition as well as the refractive index of its environment [11]. The latter feature can be exploited for biosensing as variation in the localized refractive index due to analyte interactions or conformational changes can result in LSPR peak alterations. Nanostructure LSPR has been used for several biological applications, including monitoring of protein interactions, detection of toxins, and monitoring of biomarkers, including glucose, peptides, and microRNAs [12]. Among these diverse nanostructures, TSNPs exhibit some of the strongest LSPR responsivities [13].

The extracellular matrix (ECM) is known to play an important role in regulating cell function, health, repair, and growth through a diversity of physical, chemical, and biological cues [14]. Since tissue engineering research evolved during the early 1990s, a range of 3D porous scaffolds has been developed, including biomimetic scaffolds, which have the ability to mimic the architecture of natural ECM. This is a promising technology in the advancement of tissue repair in areas including orthopedics, dermatology, and dental treatment [15]. Since tissue engineering aims to restore the tissue and organ functionality that may be lost due to injury, aging, or diseases [16], this field is indeed an advanced alternative to traditional surgical approaches of autologous and allogeneic treatment, which involve increased risks of complications such as lack of donors and a high risk of donor-site morbidity. Therefore, extensive work has been ongoing in creating scaffold formulations that have the capability to mimic the natural extracellular matrix of the body by using biodegradable materials such as chitosan [17]. An important approach involves biomimetic scaffolds suffusion with suitable growth factors to aid the process of tissue repair, such as bone tissue regeneration. The incorporated growth factors are designed to be released in a gradual manner, providing a fully functional effect on the site [18]. As such, it is important to be able to monitor the bioactivity of such growth factor through its induction of conformational changes in the cellular environment.

**Figure 1:**
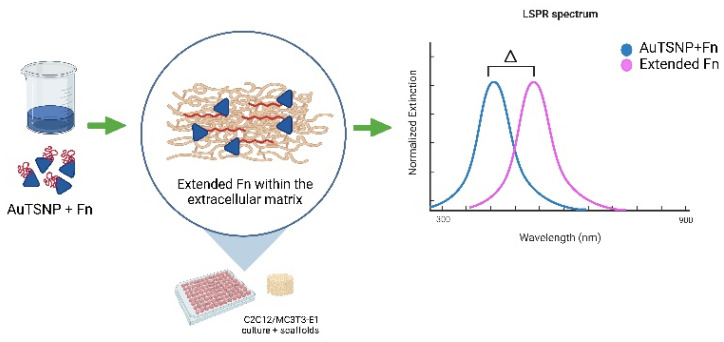
LSPR methodology for Fn extension monitoring overtime within cells ECM. Created with Biorender.com.

In the current work, an optimized formulation of high LSPR responsive gold-edge-coated triangular silver nanoparticles (AuTSNP) [19] was utilized for label-free monitoring of conformations of the ECM fibronectin (Fn) protein component in both C2C12 myoblast culture and MC3T3 pre-osteoblasts, in the presence of an optimized bioactive biomimetic bone regeneration scaffold as a model for the ECM. This LSPR methodology is illustrated in Figure 1.

## 2. Materials and Methods

High molecular weight chitosan, hydroxyapatite, tricalcium phosphate, acetic acid, reagents for AuTSNP synthesis, and fibronectin were obtained from Sigma–Aldrich. Poly (ethylene glycol) dimethyl methacrylate 600 (PEGDMA600) was obtained from Polysciences Inc. (Polysciences Europe GmbH, Germany), and Benzophenone, 99% was purchased from Alfa Aesar (ThermoFisher (Kandel) GmbH). The C2C12 myoblast cell line was purchased from the European Collection of Authenticated Cell Cultures (ECACC). MC3T3-E1 pre-osteoblast cell line (CRL-2593) was obtained from American Type Culture Collection (ATCC). Extinction measurements were performed using an Ultraviolet-visible (UV-vis) spectrometer (Synergy HT BioTek microplate reader, Winooski, VT, USA). Scaffold sterilization was performed using a pulsed UV chamber (Samtech Pulsed UV system, Ltd., Glasgow, Scotland).

### 2.1. Fabrication of Chitosan-Based Scaffolds

Chitosan (CS) scaffold composites were prepared by means of a one-step photocrosslinking method under a UV lamp as described previously [20,21]. Chitosan was first dissolved in 1% acetic acid to make 12% (*w*/*v*) chitosan paste. Following this, 0.1% (*w*/*v*) benzophenone photoinitiator and poly (ethylene glycol) dimethyl methacrylate 600 (PEGDMA600) were also added to the mixture to initiate the photocrosslinking process under UV light. Hydroxyapatite (HAp) bioceramic was incorporated into the mixture, making 1:1 CS/HAp scaffolds. The composite paste was transferred into silicone molds of 5 mm diameter and placed in a UV chamber (Dr. Gröbel UV-Electronik GmbH) with 20 UV lamps to cure for 10 min, with flipping mid-curing.

### 2.2. Scaffold Characterization

The linkage and structural properties of CS/HAp scaffolds were examined by using attenuated total reflectance Fourier-transform infrared (FTIR) spectroscopy on a Perkin–Elmer Spectrum One FTIR spectrometer fitted with a universal ATR sampling accessory. All tests were run by using a spectral range of 4000 to 650 cm^−1^. Four scans per sample cycle were utilized with a resolution of 0.5 cm^−1^ at room temperature. The samples were dried in a vacuum oven at 37 ℃ and 70 mbar prior to the tests to avoid shadowing the significant signature peaks of the materials by the broad water peaks. Following the tests, all spectra obtained were analyzed to observe the linkages formed within all the formulations.

### 2.3. Gold-Edge Coated Triangular Silver Nanoplates (AuTSNP) Preparation

Triangular silver nanoplates (TSNP) were prepared through a seed-silver catalyzed reduction of silver nitrate by sodium borohydride. The preparation of the seeds involved a one-pot synthesis in 4.5 mL of Sigma water followed by the addition of trisodium citrate (TSC) (500 μL, 25 mM), poly (4-styrene sulfonate sodium salt) (PSSS) (250 μL, 500 mg/L), sodium borohydride (NaBH_4_) (300 μL, 10 mM), and finally the addition of silver nitrate (AgNO_3_) (5 mL, 0.5 mM). The latter was added via a pump at a rate of 2 mL min^−1^. Upon seeds synthesis, TSNP production was performed by mixing 75 μL of ascorbic acid (10 mM) with 350 μL of seed solution, 4 mL of water and 3 mL of AgNO_3_ (0.5 mM) added via a pump at a rate of 1 mL min^−1^. After TSNP growth, 500 μL of TSC (25 mM) was added to the solution as a stabilizer.

TSNP were protected against etching through the deposition of a thin layer of gold on the edge of the nanoplates. This was achieved by mixing 20 μL of Gold (III) chloride trihydrate (HAuCl_4_) (0.5 mM) and 18.9 μL of ascorbic acid (10 mM) with 1 mL of TSNP solution. Further protection of the nanoplates for the protein monitoring experiments within cell culture was carried out by adding a coat of PEG 20,000 on the nanoparticle’s surface to minimize contact between the functionalized protein and the particles to avoid influence on the protein’s performance.

### 2.4. Cell Culture

For the initial experiments, the C2C12 myoblast cell line was grown in phenol red-free DMEM (Dulbecco’s Modified Eagle Medium) completed with 10% fetal bovine serum (FBS), 5% penicillin–streptomycin, and 5% L-glutamine in a humidified atmosphere (5% CO_2_ at 37 °C). C2C12 cells were plated in a 96-well plate at an initial density of 5 × 10^4^ cells per well and incubated until confluent for 24 h. Smaller diameter CS-HAp scaffolds (0.5 cm) were placed in the wells before adding the cells. PEG-Np (600 μL) were preincubated with 20 μL of fibronectin (1 mg/mL) in its active and denatured forms. Fibronectin denaturation was carried out by heating the protein in a water bath at 95 °C for 15 min. Before adding the nanoplate treatments to the cells after 24 h of incubation, the growth media was changed to fresh media. The cultured cells were incubated with the nanoplates and fibronectin-functionalized nanoplates in a PEG-Np–DMEM 1:1.167 ratio. The experiments with MC3T3-E1 pre-osteoblast cells were performed in a similar manner. Cells were cultured in phenol red-free Alpha-MEM (Minimum Essential Medium) containing 10% FBS, 5% penicillin–streptomycin and, 5% L-glutamine under an atmosphere of 5% CO_2_ at 37 °C. A density of 38 × 10^4^ cells per well was plated in a 24-well plate and incubated until confluent for 24 h. CS-HAp scaffolds were placed in transwell inserts after seeding the cells and placed in the wells for incubation. PEG-Np (1.2 mL) were preincubated with 40 μL of fibronectin (1 mg/mL) in its active and denatured forms. Prior to the addition of the nanoplates to the cells, the culture media was changed to fresh media. Cells were incubated with the nanoplates and fibronectin-functionalized nanoplates in a PEG-Np–DMEM 1:1.167 ratio.

### 2.5. Protein Monitoring

Upon incubation of the functionalized nanoplates with the cells, protein monitoring was performed using UV-Vis spectrometry with a Biotek Synergy HT microplate reader. UV measurements were taken at five different time points over 32 h (0 h, 3 h, 8 h, 24 h, 32 h) to observe shifts in the LSPR recordings indicating the Fn extension and fibril formation over time. The microplate reader was set for absorbance measurements from 300 to 900 nm wavelengths in 1 nm steps. CS/HAp scaffolds were removed from the wells before UV measurements.

## 3. Results and Discussion

### 3.1. Biomimetic Bone Regeneration Scaffold

A CS/HAp scaffold formulation with good biocompatible and osteoconductive properties [20,21] was selected to mimic the ECM. Its high performance in supporting the proliferation and attachment of alveolar bone-derived mesenchymal stem cells (ABMSCs) has been demonstrated. Increased osteogenic activity as indicated by increased alkaline phosphatase assay and osteogenic gene expression for the CS/HAp-treated ABMSCs has been observed compared to CS alone [22,23]. HAp presents as a versatile material for wide applications, including the fabrication of implants in ophthalmology and drug delivery devices as well as in mimicking vascularization [24].

**Figure 2:**
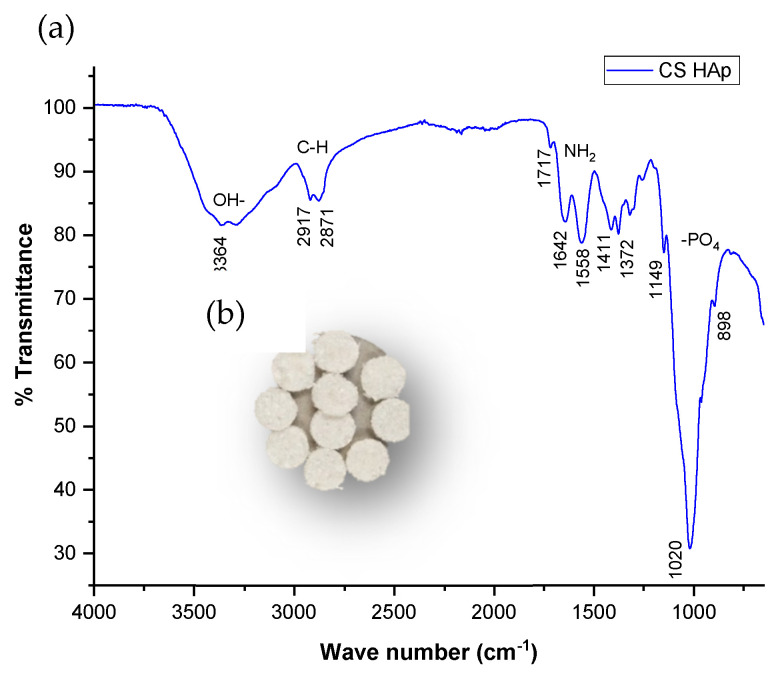
(**a**) FTIR spectrum shows the phosphate and carbonate characteristics of the bioceramics incorporated in the formulations of the scaffolds fabricated (**b**) CS/HAp scaffolds fabricated from a photocrosslinking reaction in a UV chamber.

The CS/HAp scaffolds (Figure 2b) were analyzed using FTIR to identify and confirm the incorporation of calcium phosphate materials within the scaffolds. Chitosan was characterized by peaks corresponding to N-H stretching at 1558–1642 cm^−1^ and asymmetrical C-H stretch of -CH_2_ at 2869–2921 cm^−1^ (Figure 2a) [21]. The main components of hydroxyapatite include the phosphate group (PO_3_^3-^), hydroxyl group (OH^-^), and carbonate group (CO_3_^2-^). The FTIR spectrum exhibited a phosphate band observed at 1020 cm^−1^, which was due to PO_4_ tetrahedra internal vibrations, normally seen at 1200–900 cm^−1^ [25]. The presence of the bioceramic within the scaffolds was also confirmed by orthophosphate bands visible at 1998 cm^−1^, 1030–1033 cm^−1^ (phosphate bending vibration), 960 cm^−1^ (phosphate stretching vibration), 620 and 560 cm^−1^ [26]. In addition, CO_3_^2−^ groups from the scaffolds were identified at 1411–1657 cm^−1^.

### 3.2. Fn Monitoring in C2C12 Myoblast Cells

The validation of AuTSNP as powerful biosensors for the detection of proteins and other biomolecular components in these high background noise environments is outlined. Fn behavior was monitored in the presence of cells alone, the ECM alone, and cells in the presence of the CS/HAp scaffold.

**Figure 3:**
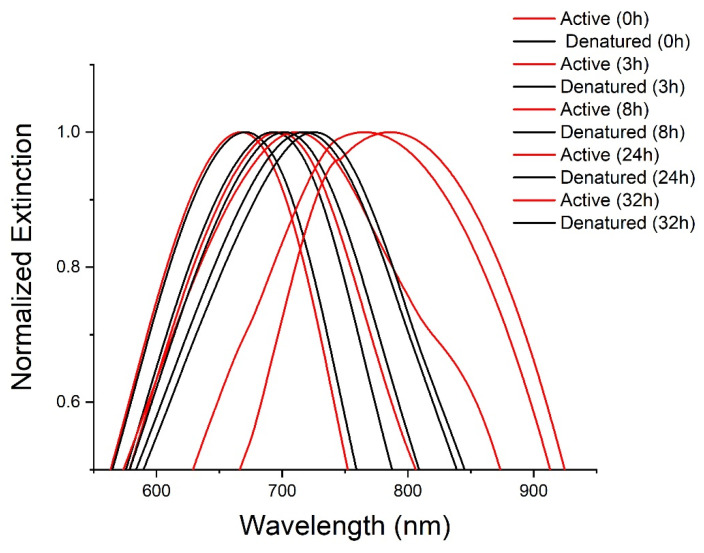
LSPR spectra of active (blue) and denatured (black) Fn PEG-Np in C2C12 cell culture from 0 h to 32 h.

C2C12 cells were incubated and treated with, functionalized AuTSNP, namely Polyethylene glycol (PEG) coated AuTSNP, denoted PEG-Np, active Fn coated PEG-Np, denoted active Fn-PEG-Np, and denatured Fn-PEG-Np as controls. Fn behavior in its active and denatured forms was analyzed over time through LSPR measurements at five different time points. PEG-Np alone was used as a further control for the impact on cellular morphology and non-specific binding [19]. Denatured Fn was used to validate the spectral red shifts recorded over time corresponding to the active Fn unfolding conformation within the ECM. As shown in Figure 3, between the 0 h and 8 h time points, a shift of ~40 nm for the active Fn was observed, while longer shifts were recorded at the 24 h and 32 h time points with shifts up to ~80 nm. Although the denatured protein showed a similar profile, shifts as short as 6 nm showed beyond the 24 h time point, which indicates the limited shifting of an inactive protein due to decreased extension activity (Table 1). Long spectral shifts for the active protein can be correlated with the Fn unfolding behavior within the extracellular matrix mediated by cells through integrin binding, which was reported as the indication of matrix aging and maturation [27,28].

**Figure 4:**
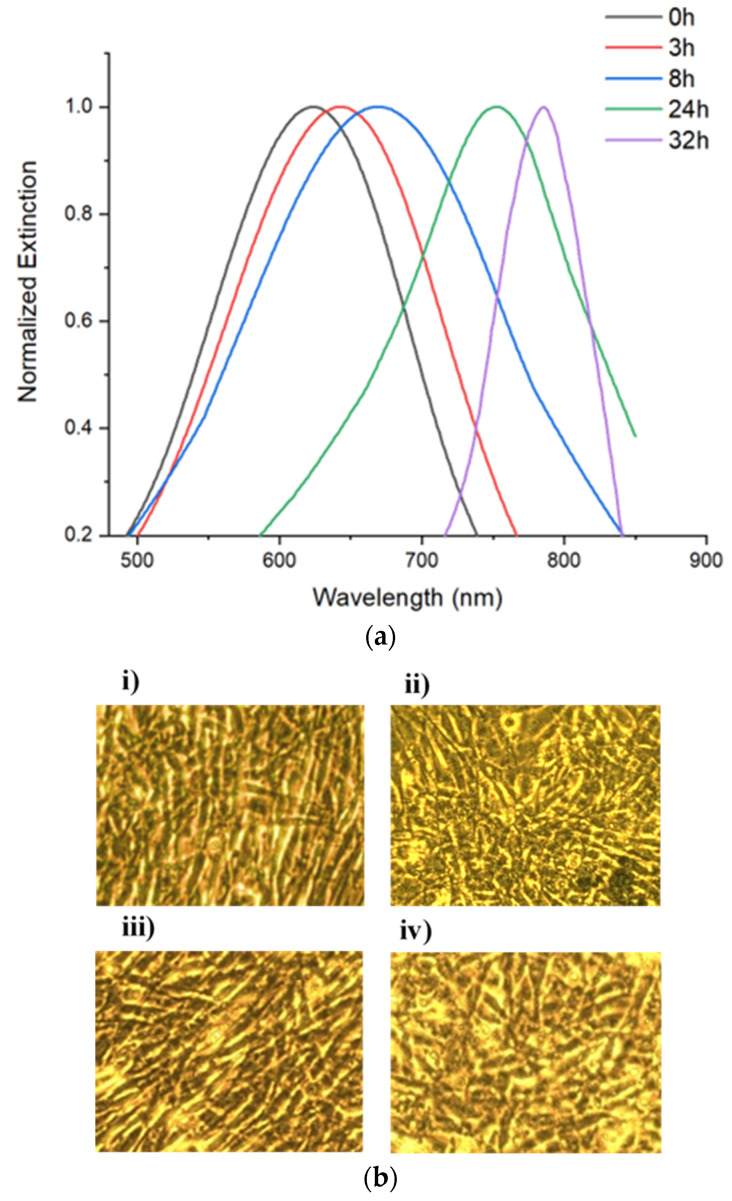
(**a**) Uv-vis spectra of Fn-PEG-Np incubated with C2C12 cells (20X magnification) in the presence of CS-HAp tissue regeneration scaffolds. (**b**) Images of C2C12 myoblast cells taken after 32 h of incubation with (**i**) PEG-Np, (**ii**) Denatured Fn-PEG-NP, (**iii**) Active Fn-PEG-NP, (**iv**) Fn-PEG-Np and scaffold.

When in presence of a CS-HAp scaffold, active Fn showed similar red-shifting performance (Figure 4a) with shifts as long as 80 nm between the 8 h and 32 h time points (Table 2).The morphology of C2C12 cells was observed to be normal with a high percentage of confluence, which indicated that the NPs had no observable negative impact on the cell culture regardless of the treatment up to 32 h (Figure 4b). This result is in keeping with findings in the literature, which reports that C2C12 cells exposed to low concentrations of silver nanoparticles show no negative effect on morphology or cytotoxicity between 0.25 and 2 ug/mL [29].

### 3.3. Fn Monitoring in MC3T3 Pre-Osteoblast Cells

**Figure 5:**
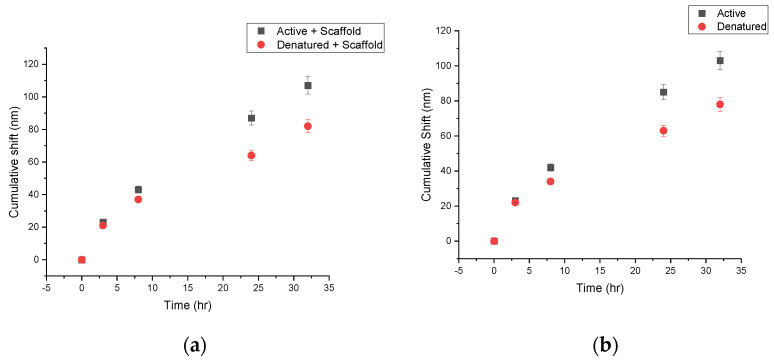
Cumulative shifts for active and denatured Fn PEG-NP within MC3T3-E1 cell culture with (**a**) and without (**b**) CS-HAp bone regeneration scaffolds.

Fn behavior was then studied in the presence of MC3T3-E1 pre-osteoblasts to observe any contrasting interactions within a different cellular environment. In these experiments, the same controls were used but with the addition of BSA-PEG-Np as a control for nanoparticle degradation due to cellular conditions and non-specific binding interactions [19]. Similar performance of the protein on and off-scaffold was observed over time with longer red shifts for the active protein in comparison to the denatured Fn. Shifts up to 103 nm can be observed by the end of 32 h for the active protein, while the inactive protein shows shorter shifts of 78 nm (Figure 5a).

It was previously reported that the development of the Fn matrix within the ECM develops after 4 h once Fn is added to the cell culture, and the most unfolded matrix is measured at the 24 h time point [27]. This correlates to the longer shifts being recorded after the 3 h time point, with the longest shifts overall occurring at 24 h (Figure 5b).

**Figure 6:**
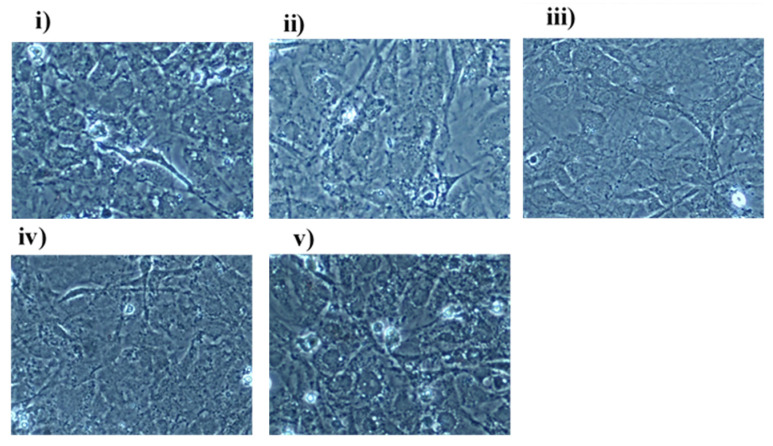
Images of MC3T3-E1 cells (20X magnification) taken after 32 h of incubation with (**i**) Active Fn-PEG-Np, (**ii**) Denatured Fn-PEG-NP, (**iii**) Active Fn-PEG-Np and scaffold, (**iv**) Denatured Fn-PEG-Np and scaffold, (**v**) BSA-PEG-NP.

Similar to previous experiments, Fn monitoring in the presence of chitosan-based scaffolds showed comparable results where longer shifts for the active Fn could be observed in comparison to shorter shifts from the denatured protein (107 nm and 82 nm respectively). Pre-osteoblast morphology and confluence can still be observed after 32 h of culture, demonstrating the cellular compatibility of the scaffold in the presence of MC3T3 cells and no observable negative effect of PEG-NP on the cells (Figure 6), as also demonstrated by Hashimoto, et al. (2013), where water-dispersible silver nanoparticles were co-cultured with the MC3T3 cell line with no cytotoxic effects observed [30].

Our results showed a similar performance to the work of Y. Zhang, et al. (2014), where TSNP were used for the detection of C-reactive protein (hs-CRP) at different concentrations, with a red-shifting profile observed as a result of the increment in the refractive index surrounding the nanoparticles as the concentration of hs-CRP increases in the samples [9]. In the case of fibronectin, red shifts were observed over time as the protein extended and interacted within the extracellular matrix of the cells, causing an increase in Fn strands that interacted with the nanoparticle surface.

For the past few years, metallic nanoparticles have been used in the biomedical field for a range of applications, such as the detection of specific antibodies and other proteins, among others. Gold nanoparticles have mostly been the gold standard for these applications due to their functional properties and straightforward synthesis; nonetheless, triangular silver nanoparticles have been reported to possess some of the highest sensitivities among noble metals and promising plasmonic properties due to their sharp corners, making them excellent alternatives for accurate biosensing applications [13,31].

## 4. Conclusions

In this work, we have validated the functionality of AuTSNPs to perform as biosensors in the presence of high-noise cellular environments, with and without the presence of ECM-mimicking bone regeneration scaffolds. The specificity of the Fn monitoring was confirmed through the implemented denatured and active protein approaches, where it was successfully demonstrated that gold edge-coated triangular silver nanoparticles are powerful tools for straightforward, versatile non-labeling measurements for biomolecule dynamics in high background noise environments such as MC3T3 and C2C12 cell lines. Results showed a highly similar shifting profile in all experiments regardless of the cultured cell line and the presence of scaffolds. This remarkable sensitivity in the presence of both cell lines and scaffold demonstrated the clear capability of the functionalized AuTSNPs in interacting and sensing tissue molecular signaling and therefore has the potential to provide extraordinary possibilities for the development and progression of regenerative medicine. Furthermore, it can provide fast and accurate detection of biomolecules of clinical importance within point-of-care clinical settings, which will lead to a better course of treatments for rising-disease challenges.

## Figures and Tables

**Table 1:** C2C12 cell culture wavelength shift table for active and denatured Fn PEG-NP.

Time	Wavelength	
	Active	Denatured
0 h	667	670
3 h	697	693
8 h	711	702
24 h	766	718
32 h	786	726

**Table 2:** C2C12 cell culture wavelength shift table for Fn PEG-NP in the presence of Cs-HAP tissue regeneration scaffolds.

Sample	Peak Wavelength (nm)
0 h	623
3 h	643
8 h	669
24 h	753
32 h	785

## Data Availability

Not applicable.
